# Owning a Pet Is Associated with Changes in the Composition of Gut Microbiota and Could Influence the Risk of Metabolic Disorders in Humans

**DOI:** 10.3390/ani11082347

**Published:** 2021-08-09

**Authors:** Javier Arenas-Montes, Pablo Perez-Martinez, Cristina Vals-Delgado, Juan Luis Romero-Cabrera, Magdalena P. Cardelo, Ana Leon-Acuña, Gracia M. Quintana-Navarro, Juan F. Alcala-Diaz, Jose Lopez-Miranda, Antonio Camargo, Francisco Perez-Jimenez

**Affiliations:** 1 Lipids and Atherosclerosis Unit, Internal Medicine Unit, Reina Sofia University Hospital, 14004 Cordoba, Spain; ja.aremon@gmail.com (J.A.-M.); pablopermar@yahoo.es (P.P.-M.); cristina12bioq@gmail.com (C.V.-D.); juanluroca855@gmail.com (J.L.R.-C.); malenipc023@gmail.com (M.P.C.); analeoaa@hotmail.com (A.L.-A.); graciaquintana@hotmail.com (G.M.Q.-N.); jfalcala@gmail.com (J.F.A.-D.); md1lomij@uco.es (J.L.-M.); 2 Department of Medicine (Medicine, Dermatology and Otorhinolaryngology), University of Cordoba, 14071 Cordoba, Spain; 3 Maimonides Biomedical Research Institute of Cordoba (IMIBIC), 14004 Cordoba, Spain; 4 CIBER Fisiopatologia de la Obesidad y Nutricion (CIBEROBN), Instituto de Salud Carlos III, 28029 Madrid, Spain

**Keywords:** gut microbiota, dysbiosis, pet, dog, cardiometabolic diseases

## Abstract

**Simple Summary:**

Metabolic syndrome represents a multicomponent disorder characterized by abdominal obesity, dyslipidemia, hypertension and impaired insulin sensitivity, which is associated with an increased risk of cardiovascular disease. The etiology of metabolic syndrome is the result of a complex interaction between genetic, metabolic and environmental factors. However, the relationship between the risk of suffering metabolic syndrome and owning pets has not been sufficiently studied, although being in contact with pets has been considered a protective factor against cardiovascular disease. Moreover, some evidence suggests that this protection might be due to favorable changes in the intestinal microbiota. Bearing this background in mind, in this work we hypothesized that people who live with pets harbor a different microbiota to those who do not own a pet, and this fact could reduce the risk of suffering metabolic syndrome.

**Abstract:**

Pet ownership positively influences clinical outcomes in cardiovascular prevention. Additionally, cardiovascular disease (CVD) has been previously linked to microbiota dysbiosis. We evaluated the influence of owning a pet and its relationship with the intestinal microbiota. We analyzed the gut microbiota from 162 coronary patients from the CORDIOPREV study (NCT00924937) according to whether they owned pets (*n* = 83) or not (*n* = 79). The pet-owner group was further divided according to whether they owned dogs only (*n* = 28) or not (*n* = 55). A 7-item pet-owners test score was used. Patients who owned pets had less risk of metabolic syndrome (MetS) (OR = 0.462) and obesity (OR = 0.519) and were younger (*p* < 0.001) than patients who did not own pets. Additionally, patients who owned dogs had less risk of MetS (OR = 0.378) and obesity (OR = 0.418) and were younger (*p* < 0.001) than patients who did not own pets. A preponderance of the genera *Serratia* and *Coprococcus* was found in the group of owners, while the genera *Ruminococcus*, an unknown genus of *Enterobacteriaceae* and *Anaerotruncus* were preponderant in the group of non-owners. In patients who owned dogs, *Methanobrevibacter* and two more genera, *Coprococcus* and *Oscillospira*, were more common. Our study suggests that the prevalence of MetS and obesity in CVD patients is lower in pet owners, and that pet ownership could be a protective factor against MetS through the shaping of the gut microbiota. Thus, owning a pet could be considered as a protective factor against cardiometabolic diseases.

## 1. Introduction

The One Health concept has been established in recent years to highlight the interaction between people, animals and the environment, reaching with increasing repercussions into the field of public health. Thus, the World Small Animal Veterinary Association has proposed interdisciplinary collaborations to promote the health of people, animals and the environment, and the health benefits of people when interacting with companion animals, as well as translational research and comparative clinics in pets, seeking the benefits of animal and human (One) health as key objectives [1].

Cardiovascular disease (CVD) is currently a major world-wide epidemic, and is associated with type 2 diabetes mellitus (T2DM) and metabolic syndrome (MetS) [2]. Several epidemiological conditions, such as age, ethnicity, gender, diet, physical activity, amongst others, have been linked to MetS [3]. However, the relationship between the risk of suffering it and owning pets has not been sufficiently studied, although being in contact with pets has been considered a protective factor against CVD [4,5] and other diseases in children, including allergies and obesity [6,7]. In fact, some evidence suggests that this protection might be due to favorable changes in the intestinal microbiota.

The gut microbiota is now recognized as an organ which is fully integrated in the metabolism of the host [8]. Recently, it has been proposed that dysbiosis may trigger the development of metabolic diseases such as obesity, MetS and T2DM [9,10,11]. Moreover, contact with pets, and particularly dogs, has been linked with changes in the gut microbiota composition, in relation to microbiota transfer and lifestyle habits [6,7]. In fact, it has even been proposed that pets and their owners share common intestinal bacteria [12].

Bearing this background in mind, we put forward the hypothesis that people who live with pets harbor a different microbiota to those who do not own a pet, and this fact could reduce the risk of suffering MetS and obesity. Thus, we aimed to evaluate the association between owning pets and the prevalence of MetS, and to explore if the microbiota composition was different between pet owners and those who do not own a pet. We therefore compared the composition of the human gut microbiota in both situations, in addition to the metabolic trait characteristics of metabolic syndrome, which are also related to cardiovascular disease.

## 2. Methods

### 2.1. Study Participants

We conducted this work within the framework of the CORDIOPREV study (Clinical Trials.gov.Identifier: NCT00924937), an ongoing prospective, randomized, open, controlled trial of 1002 patients receiving conventional treatment for coronary heart disease (CHD), who had their last coronary event over six months before enrolment in one of two different healthy dietary models (a Mediterranean (MED) diet and a low-fat (LF) diet) over a period of seven years.

The sample size was calculated following the method by Frieman et al. 1978 [13]. The proportion of the main variable studied (MetS) was stated as 58%, as in previous results of CORDIOPREV [14]. Accepting an alpha risk of 0.05 and a beta risk of 0.1 in a two-sided test, 127 subjects were required in the observed group to recognize a difference greater than or equal to 15%. We randomly selected a total list of 200 patients, on which we performed a 7-item questionnaire to evaluate pet ownership, from which a further 38 were discarded because no feces samples were available, or they had consumed antibiotics within 1 month preceding the sample collection. Therefore, we analyzed the baseline fecal samples of 162 patients (133 men and 29 women). The patients were divided into two groups, according to whether they owned a pet (pet-owner group) or not (non-pet-owner group): the former consisted of 83 patients (72 men and 11 women), while the latter was made up of 79 patients (61 men and 18 women). The metabolic characteristics of the subjects in the study are shown in Table 1. In addition, we divided the pet owners into one subgroup (dog owners), according to whether they owned dogs only (24 men and 4 women).

### 2.2. Diet Assessment

Adherence to the Mediterranean diet (MED) was assessed by a validated 14-item questionnaire [15] and to the low-fat (LF) diet by a 9-point score. This was performed once before the start of the dietary intervention and then yearly. We used Spanish food composition tables and a validated food frequency questionnaire [16] to calculate the intake of fiber.

### 2.3. Assessment of Pet Ownership

We performed a 7-item questionnaire to evaluate pet ownership, including the kind and the number of pets, the length of time they had been living with their pets, and whether they kept the pets at home or outdoors. We evaluated the ownership of dogs, cats, birds and any other animals that owners considered as a pet (included in the questionary as “others”). In the case of patients who did not own a pet, we asked whether they had previously owned a pet, and, if so, the number of pets they had had, the number of years they had been living with them, and the time elapsed since they last lived with a pet (Table S1).

### 2.4. Clinical Plasma Parameters

To collect blood samples, we used tubes containing 0.1% EDTA, which were then centrifuged at 1500× *g* for 15 min at 4 °C to separate the plasma from the blood cells. From frozen samples, blinded to the team members, analytes were measured at the Lipid and Atherosclerosis Unit at Reina Sofia University Hospital by members of the laboratory research team, as previously described [17].

### 2.5. DNA Extraction from Fecal Samples

We gave the patients a box with carbonic ice and a sterile plastic bottle with a screw cap to collect fecal samples. This allowed us to keep the samples frozen after delivery to the laboratory staff and store them at −80 °C. DNA was extracted using the QIAamp DNA Stool Mini Kit (Qiagen, Hilden, Germany), following the manufacturer’s instructions. DNA samples were stored at −20 °C, after quantification with the Nanodrop ND-1000 v3.5.2 spectrophotometer (Nanodrop Technology^®^, Cambridge, UK), as previously described [17].

### 2.6. Sequencing and Bioinformatics

For each DNA (fecal) sample, we amplified by polymerase chain reaction the hypervariable regions V3 and V4 of the 16S rRNA gene using the primer pair 5′-TCGTCGGCAGCGTCAGATGTGTATAAGAGACAG-3′ and 5′-GTCTCGTGGGCTCGGAGATGTGTATAAGAGACAG-3′ [18], which was further sequenced on a MiSeq Illumina platform (Illumina, San Diego, CA, USA). Briefly, PCRs were performed using a KAPA HiFi HotStart ReadyMix (KAPABIOSYSTEMS), 1.25 μL of extracted DNA (5 ng/μL in 10 mM Tris pH8.5) and 0.2 μM of each primer, using the following cycle parameters: 3 minutes denaturation at 95 °C, followed by 25 cycles (30 s at 95 °C, 30 s at 60 °C, 30 s at 72 °C) and a final extension at 72 °C for 5 min. The 16S V3 and V4 amplicon purification was performed using Agentcourt AMPure XP beads (Beckman Coulter). A second PCR attached dual indices and Illumina sequencing adapters using the Nextera XT Index Kit. This PCR was performed with a KAPA HiFi HotStart ReadyMix (KAPABIOSYSTEMS), with 5 μL of the previous amplicon, 5 μL of each Nextera XT Index Primer 1(N7xx) and 5 uL of each Nextera XT Index Primer 2(S5xx), using the following cycle parameters: 3 min denaturation at 95 °C, followed by 8 cycles (30 s at 95 °C, 30 s at 55 °C, 30 s at 72 °C), and a final extension at 72 °C for 5 min, as previously described [19]. Sequence outputs were analyzed using the Quantitative Insights into Microbial Ecology (QIIME) program, version 1.9.1 [20], using QIIME default parameters. The 16S paired reads were assembled using the script multiple_join_paired_ends.py, which joins forward and reverse demultiplexed reads. The output file was processed for quality filtering by split_libraries_fastq.py. High quality sequences were grouped into Operational Taxonomic Units (OTUs) with a sequence identity threshold of 97%, and taxonomy was assigned by interrogating the high quality sequences with the Greengenes database (13_5) [21]. Bacterial richness and diversity across the samples were calculated using the Chao1, Simpson, and Shannon indexes [22]. Linear discriminant analysis (LDA) effect size (LEfSe) (http://huttenhower.sph.harvard.edu/galaxy/ (accessed on 14 April 2020)) was used to compare groups at baseline and visualize the results using taxonomic bar charts and cladograms [23].

### 2.7. Data Accesibility

The sequences obtained in this study have been submitted to NCBI Sequence Read Archive (SRA) under the accession number PRJNA612957 (https://www.ncbi.nlm.nih.gov/sra/PRJNA612957).

### 2.8. Statistical Analysis

The PASW statistical software package, version 20.0 (IBM Inc., Chicago, IL, USA), was used for univariate statistical analyses of data. A *p*-value less than 0.05 was considered significant. The normal distribution of variables was assessed using the Kolmogorov–Smirnov test. Statistical differences in the main metabolic variables between the groups were evaluated using one-way ANOVA tests. All the quantitative data shown in this study were expressed as mean ± standard deviation (SD). The Odds Ratio (OR), a measure of association that represents the odds that an outcome will occur given a particular exposure, compared to the odds of the outcome occurring in the absence of that exposure [24], was also obtained to calculate the risk of each group.

## 3. Results

### 3.1. Baseline Characteristics of the Study Participants

The differences in the main dietary, anthropometric and metabolic variables between groups are shown in Table 1. No statistically significant differences were observed in the main dietary and metabolic variables, but patients who owned a pet were younger than patients who did not (*p* < 0.001). In addition, patients who owned a pet had less risk of MetS (OR = 0.241 − **0.462** − 0.883; *p* = 0.01) and obesity (OR = 0.278 − **0.519** − 0.971; *p* = 0.03) than patients who did not. Moreover, differences between dog owners and non-pet owners are shown in Table 2. Dog owners were younger (*p* < 0.001) and had less risk of MetS (OR= 0.144 − **0.378** − 0.991; *p* = 0.04) and obesity (OR= 0.173 − **0.418** − 1.010; *p* = 0.0496) than patients who did not own a pet. The pets owned by participants were 56 dogs, 19 cats, 35 birds, and 11 pets classified as “others” (including foxes, turtles, fishes, etc.). Please note that a person could simultaneously own different species. Three pets had chronic diseases, three pets had any remarkable disease and four pets usually had digestive problems (diarrhea, constipation, vomiting).

### 3.2. Microbiota Characteristics of the Study Participants

**Figure 1:**
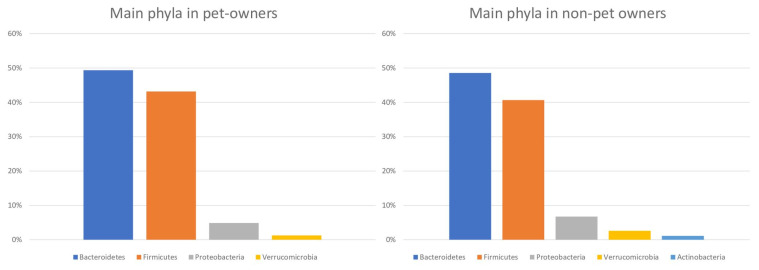
Each bar represents the mean relative sequence abundances of the phyla (only those phyla with an abundance higher than 1% are included).

In the pet-owners fecal microbiota samples, four phyla had relative sequence abundances greater than 1%: *Bacteroidetes* (49.33%), *Firmicutes* (43.16%), *Proteobacteria* (4.85%) and *Verrucomicrobia* (1.21%). In the non-pet-owner gut microbiota samples, five phyla had relative abundances greater than 1%: *Bacteroidetes* (48.54%), *Firmicutes* (40.65%), *Proteobacteria* (6.70%), *Verrucomicrobia* (2.58%), and *Actinobacteria* (1.11%) (Figure 1). The fecal microbiota samples from pet owners revealed that the bacterial genera with a >1% abundance were *Bacteroides* (24.28%), *Prevotella* (11.60%), an unknown genus of *Ruminococcaceae* (9.08%), an unknown genus of *Clostridiales* (7.93%), an unknown genus of *Lachnospiraceae* (3.74%), *Parabacteroides* (3.58%), *Ruminococcus* (3.02%), *Phascolarctobacterium* (2.91%), *Faecalibacterium* (2.78%) and *Lachnospira* (2.78). By contrast, the intestinal microbiota samples of patients who did not own a pet showed that bacterial taxa with a >1% abundance were *Bacteroides* (27.64%), *Prevotella* (8.52%), an unknown genus of *Ruminococcaceae* (7.76%), an unknown genus of *Clostridiales* (6.37%), *Parabacteroides* (4.10%), an unknown genus of *Lachnospiraceae* (3.34%), an unknown genus of *Enterobacteriaceae* (3.33%) *Lachnospira* (2.92%), *Phascolarctobacterium* (2.85%), *Ruminococcus* (2.84%) and *Akkermansia* (2.58%).

### 3.3. Differences in the Gut Microbiota between Pet Owners and Non-Pet Owners: LEfSe Analysis

**Figure 2:**
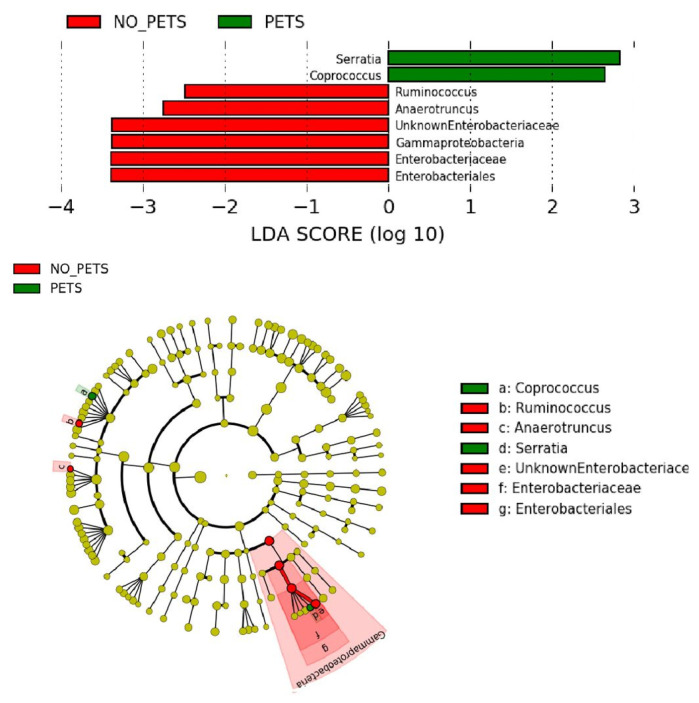
Cladogram representing the taxonomic hierarchical structure of the identified differences between pet owners and non-pet owners using Linear discriminant analysis effect size. Each filled circle represents one phylotype. Red, bacterial taxa statistically overrepresented in non-pet owners; green, bacterial taxa overrepresented in pet owners. Phylum and class are indicated in their names on the cladogram and the order, family, or genus are given in the key.

In order to evaluate changes in the human gut microbiota due to pet ownership, we assessed the global differences between patients who owned pets or did not. We used LEfSe to compare the estimated phylotypes between the groups and, as can be seen in Figure 2, a preponderance of *Serratia* and *Coprococcus* was significant in the owners while the non-owners had a preponderance of one genus of the *Gammaproteobacteria* class from the *Enterobacteriaceae* family, and two genera of the *Clostridiales* order, namely *Ruminococcus* and *Anaerotruncus*.

### 3.4. Differences in the Gut Microbiota between Dog Owners and Non-Pet Owners: LEfSe Analysis

**Figure 3:**
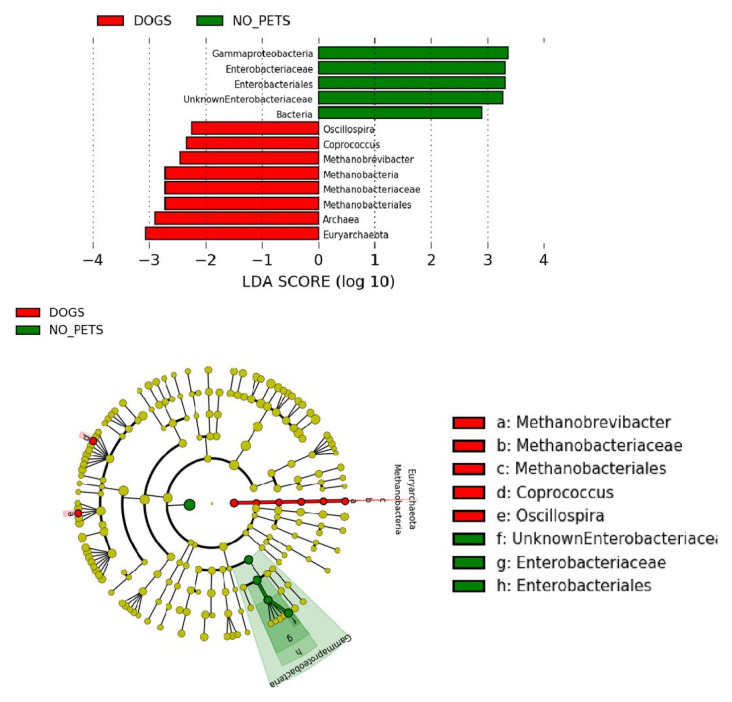
Cladogram representing the taxonomic hierarchical structure of the identified differences between dog owners and non-pet owners using Linear discriminant analysis effect size. Each filled circle represents one phylotype. Red, bacterial taxa statistically overrepresented in dog owners; green, bacterial taxa overrepresented in non-pet owners. Phylum and class are indicated in their names on the cladogram and the order, family, or genus are given in the key.

To discern whether these differences in patients who owned pets were specific to dog owners, which were the majority group in the pet owners, we compared dog owners with non-pet owners (Figure 3). We used LEfSe to compare the estimated phylotypes of these groups. The dog owners’ gut microbiota was characterized by a preponderance of the domain *Archaea*, one genus of the *Methanobacteriales* class, *Methanobrevibacter*, and two more genera, *Coprococcus* and *Oscillospira*, whereas the non-pet owners’ intestinal microbiota was characterized by a preponderance of the domain *Bacteria* and an unknown genus of the *Enterobacteriaceae* family from the *Gammaproteobacteria* class.

## 4. Discussion

This work provides evidence on the existence of specific differences in the intestinal microbiota composition according to whether the patient owns a pet or not, and its potential association with MetS. Moreover, we identified specific gut microbiota features associated particularly to owning a dog, and that pet ownership was linked to a lower risk of MetS and obesity.

Currently, the relationship between microbiota and disease is an emerging field in research. Studies in humans have identified a direct interaction between microbiota dysbiosis and the incidence of diseases such as nonalcoholic fatty liver disease and inflammatory bowel disease [25,26]. In addition, microbiota dysbiosis has also been related to a higher incidence of metabolic diseases and CVD, including MetS and obesity [27,28,29].

Previous data have indicated that factors, such as owning a pet, seem to affect the gut microbiota composition [30]. Additionally, it has also been described that pet ownership is associated with a lower risk of suffering CVD, mainly by providing social support and motivation for physical activity [4,5]. Despite this increasing knowledge, the potential improvement in the gut microbiota profile associated with pet ownership in cardiovascular disease patients has not yet been studied. In addition, to the best of our knowledge, none of the published studies have investigated the relationship between pet ownership and MetS.

It is interesting to note that differences in the gut microbiota in pet owners have been linked as a protective factor against the development of diseases such as allergies and obesity in infants [6,7]. In line with this, our results provide evidence that, in cardiovascular disease patients, pet owners and dog owners may have less risk of MetS than non-pet owners (OR = 0.1 − **0.42** − 0.94). Despite the difference in MetS prevalence between these groups, several MetS-related parameters, such as triacylglyceride levels and blood pressure were not statistically significant different, but were higher in patients with no pets; whereas, no differences were found in parameters such as HDL, presumably as a consequence of the differences in the HDL levels between sexes for this variable. In addition, although not statistically significantly different, others parameters such as LDL related to CVD, and CRP related to the inflammatory status, and in turn CVD, were higher in patients with no pets. Taken together, these observations suggest a better cardiometabolic status of pet owners and dog owners versus non-pet owners.

Furthermore, there was a small age difference between groups (5 years), which we did not consider relevant to influencing our findings. In addition, MetS and age were not statistically related in the study population in the comparison between the groups of pet owners and non-pet owners, and in the comparison between the groups of dog owners and non-pet owners.

Moreover, our study also showed that this difference in MetS prevalence between pet owners and the group with no pets was accompanied by differences in the gut microbiota. In fact, compared to the group of pet owners, the group with no pets was characterized by a gut microbiota with a preponderance of *Ruminococcus*, *Anaerotruncus* and an unknown genus of *Enterobacteriaceae*. The abundance of *Ruminococcus* and *Anaerotruncus* in the human microbiota has been previously related to a higher prevalence of MetS [19,31] and the abundance of *Enterobacteriaceae* has been positively linked to the development of obesity in children and pregnant women [32,33,34].

In our study, the gut microbiota of pet owners was characterized by higher levels of *Coprococcus* and *Serratia*. The genus *Coprococcus* has been shown to be a protective factor against MetS and T2DM [35] and is a short-chain fatty acid producer that modulates insulin resistance [36]. Although it is not a pathogenic genus, lower levels of *Coprococcus* are strongly associated with fasting serum levels of glycerol, monounsaturated fatty acids and saturated fatty acids, and inversely associated with polyunsaturated fatty acids [37]. *Serratia*, on the other hand, is a potentially pathogenic genus; however, its presence in healthy people has been related to it being a protective factor against obesity [38]. Thus, our findings are in line with previous studies describing an altered abundance of these bacterial taxa in metabolic disease, as the group of pet owners had less risk of MetS and obesity.

Considering the potential of different animals to change the human gut microbiota [6,7,30], and particularly the fact that they have been shown to be a protective factor against various diseases such as allergies [39], we explored the specific bacterial differences between the subgroup of patients who only owned dogs, versus the non-pet owners in general. Here, our data showed that the genera *Oscillospira*, *Coprococcus* and *Methanobrevibacter*, together with the *Archaea* domain, were found in higher proportions in the gut microbiota of dog owners. The higher prevalence of *Archaea* is probably due to the higher proportion of *Methanobrevibacter*, an interesting methanogen genus and SCFA producer [40] which has been linked to a lower body mass index (BMI) and lower levels of triglycerides, while a lower fecal concentration of this genus has been found in prediabetic subjects [37].

In addition, the *Oscillospira* genus has been correlated with leanness, lower BMI and a lower prevalence of obesity, and is able to degrade host glycans (such as fucose, sialic acids and glucuronic acid) [41,42].

In agreement with these findings, the increased presence of *Oscillospira*, *Methanobrevibacter and Coprococcus* in the samples of dog owners could explain the lower prevalence of MetS and obesity in this population [35,36,37]. These genera are not related to human infections. Very few studies have explored the gut microbiota of people who come into contact with pets, and in all of them, infants were studied. However, these studies have shown that two of the four genera that our data showed as more prevalent in pet owners and dog owners, *Coprococcus* and *Oscillospira*, have been previously linked to pet ownership, which reinforces the hypothesis of the transfer of microbiota from pets to owners. In fact, higher levels of *Coprococcus* and *Oscillospira* have been previously linked to infants who live in a household where there is a dog [6,7].

By contrast, the other two genera, *Serratia* and *Methanobrevibacter*, have not yet been reported as being higher in humans who have contact with pets. Even though *Serratia* has not been identified in humans in contact with pets, this genus has been found in healthy dogs, which constitute the majority of the pets owned by our patients, which suggests that it could be transferred from pets to their owners [43,44]. Like *Serratia*, a higher abundance of *Methanobrevibacter* has not been found previously in humans living with dogs; however, this genus can also be present in the dog microbiota [45].

Diet consumption is an important factor that can modulate the intestinal microbiota. Specifically, a LF diet has been related to an increased proportion of the Prevotella genus and F. prausnitzii genera and a decreased proportion of the Roseburia genus, whereas the MED diet has been related to decreased levels of the Prevotella genus and increased levels of P. distasonis, Roseburia and Oscillospira genera [46]. However, from these, only Oscillospira has been related to pet ownership [7], and no other differences in these genera were found between groups, presumably because of the fact that there were not statistical differences in diet consumption between the groups of pet owners.

Our study has limitations and could be considered a preliminary study. One limit lies in the fact that the population in which we performed the study has CVD and may already present several potential alterations in their gut microbiota associated with this disease. Further studies are needed to clarify the relationship between cardiovascular disease and pet ownership, with particular reference to changes in the gut microbiota that may be produced as a consequence. Moreover, the study was performed in a reduced sample size population, which limits our findings. In addition, our study did not consider the age of the animals, which should also be considered in future studies. Thus, it will be necessary to replicate the study in other populations and undertake validation in a cohort without cardiovascular disease and closer to the general population.

## 5. Conclusions

Our study suggests that the prevalence of MetS and obesity in CVD patients is lower in pet owners, and that pet ownership could be a protective factor against MetS by shaping the gut microbiota. Moreover, the microbiota profile found in pet owners and dog owners was consistent with the prevalence of obesity and MetS in this population. Thus, owning a pet may be considered as a protective factor against cardiometabolic diseases.

## Figures and Tables

**Table 1:** Baseline characteristics of the participants in the study. Values correspond to the mean ± SD.

-	Patients	Pets	No Pets	*p*-Value
*n*	162	83	79	n/a
*Men/Women (n)*	133/29	72/11	61/18	0.114
*Diet (LF* vs. *MED)*	68/94	35/48 (LF vs. MED)	33/46 (LF vs. MED)	0.959
*T2DM (No* vs. *T2DM)*	49/113	27/56 (No vs. T2DM)	22/57 (No vs. T2DM)	0.517
*Metabolic syndrome (No* vs. *MetS*)	101/61	59/24 (No vs. MetS)	42/37 (No vs. MetS)	**0.019**
*Obesity (No* vs. *Obesity)*	77/85	46/37 (No vs. Obesity)	31/48 (No vs. Obesity)	**0.039**
*Arterial hypertension (No* vs. *AHT)*	53/109	33/50 (No vs. AHT)	20/59 (No vs. AHT)	0.050
*Age (years)*	63.32 ± 8.45	60.86 ± 8.21	65.92 ± 7.96	**<0.001**
*Weight (Kg)*	82.75 ± 13.35	83.15 ± 14.2	82.35 ± 12.5	0.708
*BMI (Kg/m^2^)*	30.36 ± 3.94	29.88 ± 3.88	30.85 ± 3.96	0.123
*Serum triacylglycerols (mg/dL)*	129.32 ± 88.49	115.71 ± 46.41	143.44 ± 115.89	0.237
*Total cholesterol (mg/dL)*	160.47 ± 34.24	156.87 ± 31.73	164.20 ± 36.5	0.191
*HDL-cholesterol (mg/dL)*	40.98 ± 9.64	41.29 ± 9.83	40.66 ± 9.49	0.745
*LDL-cholesterol (mg/dL)*	93.63 ± 27.92	92.02 ± 26.54	95.37 ± 29.42	0.456
*CRP (mg/dL)*	2.77 ± 3.81	2.51 ± 2.94	3.03 ± 4.54	0.106
*ISI*	4.07 ± 2.62	4.29 ± 2.86	3.80 ± 2.29	0.356
*Systolic BP*	136.55 ± 19.02	134.00 ± 17.58	139.34 ± 20.23	0.084
*Diastolic BP*	76.86 ± 11.22	77.11 ± 9.89	76.59 ± 12.59	0.776

LF, low-fat diet; MED, Mediterranean diet; T2DM, type 2 diabetes mellitus; MetS, metabolic syndrome; AHT, arterial hypertension; BMI, body mass index; CRP, C-reactive protein; ISI, insulin sensitivity index and BP, blood pressure. The statistical differences between groups were evaluated by χ2 test (men/women) or one-way ANOVA.

**Table 2:** Differences between dog-owners and non-pet owners. Values correspond to the mean ± SD.

-	Dogs	No Pets	*p*-Value
*n*	28	79	n/a
*Men/Women (n)*	24/4	61/18	0.339
*Diet (LF* vs. *MED)*	17/11 *(LF* vs. *MED)*	33/46 *(LF* vs. *MED)*	0.084
*T2DM (No* vs. *T2DM)*	8/20 *(No* vs. *T2DM)*	22/57 *(No* vs. *T2DM)*	0.942
*Metabolic syndrome (No* vs. *MetS)*	21/7 *(No* vs. *MetS)*	42/37 *(No* vs. *MetS)*	**0.044**
*Obesity (No* vs. *Obesity)*	17/11 *(No* vs. *Obesity)*	31/48 *(No* vs. *Obesity)*	**0.049**
*Arterial hypertension (No* vs. *AHT)*	10/18 *(No* vs. *AHT)*	20/59 *(No* vs. *AHT)*	0.293
*Age (years)*	60.17 ± 7.70	65.92 ± 7.96	**<0.001**
*Weight (Kg)*	81.85 ± 12.16	82.35 ± 12.50	0.860
*BMI (Kg/m^2^)*	29.40 ± 3.15	30.85 ± 3.96	0.093
*Serum triacylglycerols (mg/dL)*	115.32 ± 49.89	143.44 ± 115.89	0.218
*Total cholesterol (mg/dL)*	155.89 ± 30.53	164.20 ± 36.50	0.284
*HDL-cholesterol (mg/dL)*	40.50 ± 10.97	40.66 ± 9.49	0.777
*LDL-cholesterol (mg/dL)*	91.96 ± 24.56	95.37 ± 29.42	0.586
*CRP (mg/dL)*	2.57 ± 2.40	3.03 ± 4.54	0.608
*ISI*	4.03 ± 2.13	3.80 ± 2.29	0.715
*Systolic BP*	134.11 ± 17.48	139.34 ± 20.24	0.232
*Diastolic BP*	76.46 ± 11.60	76.59 ± 12.59	0.962

LF, low-fat diet; MED, Mediterranean diet; T2DM, type 2 diabetes mellitus; MetS, metabolic syndrome; AHT, arterial hypertension; BMI, body mass index; CRP, C-reactive protein; ISI, insulin sensitivity index and BP, blood pressure. The statistical differences between groups were evaluated by χ2 (men/women) test or one-way ANOVA.

## Data Availability

The sequences obtained in this study have been submitted to NCBI Sequence Read Archive (SRA) under the accession number PRJNA612957 (https://www.ncbi.nlm.nih.gov/sra/PRJNA612957).

## Supplementary Materials

The following are available online at https://www.mdpi.com/article/10.3390/ani11082347/s1, Table S1: A 7-item questionnaire to evaluate pet ownership, including the kind and the number of pets, the length of time they had been living with their pets, and whether they kept the pets at home or outdoors.
