# Supplementary material for: Owning a Pet Is Associated with Changes in the Composition of Gut Microbiota and Could Influence the Risk of Metabolic Disorders in Humans
Source: Animals (Basel). 2021 Aug 9;11(8):2347. doi: 10.3390/ani11082347 (PMC8388619; doi:10.3390/ani11082347)
Supplement: Supplementary file 1 [file animals-11-02347-s001.zip › animals-1300819-supplementary.pdf]

IMIBIC

NAME: ________________________________TLF:_____________ PATIENT CODE:__________

1ª. ¿Do you currently live with a pet?:

□ No. *Please go to question 8*

□ Yes, with: □ Dog □ Cat □ Birds □ Other: _____________________

2ª. How many pets do you own?: □ One □ 2-3 □ 4 or more

3ª. How long have you been living with a pet?: □ Less tan a year □ From 1 to 5 years □ More tan 5 years

4ª. Does your pet usually: ...eat with you? □ No □ Yes ...sleep with you? □ No □ Yes

5ª. Does your pet have any chronical disease? □ No □ Yes: which? _________________

6ª. ¿Has your pet had any remarkable illness? □ No □ Yes: which? ______________

7ª. Does your pet usually have digestive problems? (diarrhea, constipation, vomiting ...) □ No □ Yes

Thank you very much for your cooperation

8ª. Have you previously lived with a pet?:

□ No. *Please go to question 17*

□ Yes: □ Dog □ Cat □ Birds □ Other: _____________________

9ª. How many pets have you previously own?: □ One □ 2-3 □ 4 or more

10ª. How long have you not lived with pets?: □ <1 year □ 1-5 years □ >5 years

11ª. How long did you live with your pets□ <1 year □ 1-5 years □ >5 years □ always

12ª. Did your pets use to: ...eat with you? □ No □ Yes ...sleep with you? □ No □ Yes

13ª. ¿Do you remember if your pet had a chronic illness? □ No □ Yes: which?________

14ª. ¿Do you remember if your pet had any remarkable illness? □ No □ Yes: which? _______

15ª. Did your pet have digestive problems (diarrhea, constipation, vomiting ...)? □ No □ Yes

Thank you very much for your cooperation
